# High-precision deformation monitoring and intelligent early warning for wellbore based on BDS/GNSS

**DOI:** 10.1371/journal.pone.0325913

**Published:** 2025-06-23

**Authors:** Jiang Li, Lei Dai, Keke Xu, Xinyu Mei, Yifu Liu, Jianlin Shi, Hebing Zhang

**Affiliations:** 1 Yong Coal Company Geological Survey Department, Henan Energy Group, Yongcheng, Henan, China; 2 School of Surveying and Land Information Engineering, Henan Polytechnic University, Jiaozuo, Henan, China; ICIMOD: International Centre for Integrated Mountain Development, NEPAL

## Abstract

To address the complex deformation of wellbores influenced by surrounding coal mining operations, this study employed an improved modified least-squares ambiguity decorrelation (MLAMBDA) algorithm based on the double-difference model for high-frequency dynamic computation of Bei Dou System and Global Navigation Satellite System (BDS/GNSS) observation data. A quantitative analysis was conducted on the performance of various combinations of BDS/GNSS in wellbore deformation monitoring, and the effects of different baseline lengths on the monitoring results were evaluated. Based on the high-precision deformation monitoring sequences, an intelligent early warning model for wellbore deformation was established using the deep learning Bi-LSTM algorithm. The results indicate that the monitoring accuracy of the BDS/GNSS multi-system combination in the E, N and U directions is within 2 mm, with all three directions outperforming the results obtained from a single Global Position System (GPS) system. As the baseline length increased from 1 km to 6 km, the accuracy in the E, N, and U directions decreased by 15.8%, 16.0%, and 5.6%, respectively. Within a 6 km range, the horizontal accuracy remains better than 3 mm, while the vertical accuracy is better than 6 mm, meeting the requirements for wellbore deformation monitoring. The early warning model can flexibly adapt to the deformation conditions at different sites and the various disturbances encountered, effectively capturing the complex nonlinear time-varying characteristics of the observation time series. The prediction of future results for one month based on one year of observation sequences achieves an accuracy better than mm, providing a safeguard for safe production in mines. This research method can also be extended to use BDS/GNSS for hourly level high-precision deformation monitoring and early warning of major engineering infrastructure such as bridges, dams, and high-speed railway systems.

## 1. Introduction

As the mining depth increases, the industrial square in the mining area will inevitably experience widespread surface deformation due to the influence of deep, water-bearing loose layers. This deformation can lead to serious issues such as wall failure, tower tilt, and the risk of cage collisions. Additionally, hazards caused by wellbore deformation include water inflow, rock falls, and distortion of the cage tracks, all of which directly threaten the safety of hoisting operations and pose significant risks to the safety of personnel and property [1]. Therefore, to ensure safe production in mines, there is an urgent need to conduct deformation monitoring research on the surface of the industrial square, providing a basis for managing subsidence and predicting surface deformation. Traditional monitoring technologies, such as optical instruments or electronic devices like total stations, perform periodic point-by-point observations of the monitoring targets. These methods are often limited by visibility conditions, consume significant time and manpower, and exhibit low operational efficiency. Additionally, data processing tends to be delayed, making real-time monitoring unfeasible [2–4]. With the advancement of technology and the progression of industrial processes, deformation environments have become increasingly complex. For instance, wellbore deformation is not simply linear but exhibits nonlinear characteristics over multiple time periods. Traditional monitoring methods involve periodic observations of the wellbore and fitting deformation curves using time-averaged values, which can obscure the nonlinear changes occurring within those periods. Therefore, there is an urgent need for a comprehensive intelligent high-frequency monitoring system and high-precision data processing and analysis techniques to effectively monitor and provide early warnings for specific deformation events [5–9]. Relevant researchers have conducted many high-precision deformation monitoring studies on the surface of mining areas. The surface Global Position System (GPS) measured data of Kunyang phosphate mine and Interferometric Synthetic Aperture Radar (InSAR) monitoring data were utilized, and a Kriging interpolation method was employed to effectively fill in the missing values of incoherence, resulting in a high-precision deformation field [10].The issue of extracting noise signals in mine area GPS deformation monitoring was addressed, and a Kalman-Empirical Mode Decomposition (EMD) method was proposed, which effectively extracts the deformation signal [11]. An ambiguity fixing model with satellite and receiver Combination Phase Delay (CPD) for zero-differenced PPP ambiguity fixing was also proposed, exploring the feasibility of using PPP for deformation monitoring at four Continuous Operational Reference System (CORS) stations at the mine site [12]. The Global Navigation Satellite System (GNSS), with its advantages of high precision, high efficiency, high frequency, and real-time capabilities, can provide all-weather, continuous, and synchronized three-dimensional deformation monitoring of industrial squares and wellbore monitoring points. Notably, the completion of China’s Bei Dou System (BDS) global constellation in 2020 has further enhanced the satellite distribution and quantity available for high-precision deformation monitoring [13].

To assess the applicability and stability of BDS in large-scale infrastructure and major engineering projects, and to promote the application of the BDS industry, this study focuses on wellbore monitoring in mining areas. It examines the complex deformation conditions of mining platforms affected by surrounding coal mining activities. The advantages of BDS/GNSS multi-system combination positioning over traditional single GPS positioning are tested in terms of satellite visibility, dilution of precision (DOP) values, geometric configuration, and other aspects. Additionally, the impact of different baseline lengths on the accuracy of wellbore deformation monitoring is analyzed. The deformation monitoring of the mining area in this study was carried out slowly. We used 2880 epochs per day with a sampling interval of 15 seconds for monitoring, in order to obtain the corresponding monitoring accuracy. Finally, an intelligent early warning model for mining wellbores is established using the Bidirectional Long Short-Term Memory (Bi-LSTM) method, based on the multi-frequency and multi-mode synergy of BDS/GNSS, ensuring safe production in the mines.

## 2. Methods

Global Navigation Satellite System (GNSS) displacement monitoring is usually composed of GNSS receiver, data processing unit, data transmission module and monitoring center software. Its working principle is based on satellite signal reception and distance calculation. The GNSS receiver is responsible for capturing the signals emitted by multiple satellites (such as GPS, Beidou, etc.), which have precise time stamps. By measuring the time the signal arrives, the receiver is able to calculate the distance from the satellite, which in turn determines its accuracy three-dimensional coordinates. Changes in these coordinates are continuously monitored over time, resulting in displacement data. Differential positioning technology further improves the accuracy of monitoring results. By combining the corrected data of the reference station with known accurate position, the positioning data of the monitoring point is corrected, and the displacement monitoring accuracy of millimeter level is realized.

### 2.1. BDS/GNSS data processing strategy

Using the double-difference observation model, the equations are obtained by performing single differencing at the stations and then differencing between the satellites. Let stations  i  and  j  perform synchronous observations on satellites  s and  t . Equation (1) presents the complete double-difference observation model:


$$\lambda \nabla \Delta \phi_{ij}^{st}=\nabla \Delta \rho_{ij}^{st}-\nabla \Delta I_{ij}^{st}+\nabla \Delta T_{ij}^{st}+\lambda \nabla \Delta N_{ij}^{st}+\nabla \Delta \varepsilon_{ij}^{st}$$ (1)

where ∇Δ  represents the double-difference operator. $\rho_{ij}^{st},I_{ij}^{st},T_{ij}^{st},N_{ij}^{st},\varepsilon_{ij}^{st}$ denote distance, ionospheric delay, tropospheric delay, whole week number, and phase noise, respectively.

In real-time dynamic relative positioning, for short baselines, the two observation environments can be approximated to be the same, so that the current layer and tropospheric delay errors are negligible after double differencing. We can then rewrite equation (1) as:


$$\lambda \nabla \Delta \phi_{ij}^{st}=\nabla \Delta \rho_{ij}^{st}+\lambda \nabla \Delta N_{ij}^{st}+\nabla \Delta \varepsilon_{ij}^{st}$$ (2)

Performing linearization by Taylor series expansion for equation (2), we get the linearized form equation (3), In equation (3), $\Delta l_{j}^{st}=l_{j}^{t}-l_{j}^{s}$, $\Delta m_{j}^{st}=m_{j}^{t}-m_{j}^{s}$, $\Delta n_{j}^{st}=n_{j}^{t}-n_{j}^{s}$.


$$\lambda \nabla \Delta \phi_{ij}^{st}=[\begin{array}{ccc} \Delta l_{j}^{st} & \Delta m_{j}^{st} & \Delta n_{j}^{st} \end{array}][\begin{array}{c} \Delta X\\ \Delta Y\\ \Delta Z \end{array}]-\lambda \nabla \Delta N_{ij}^{st}+\nabla \Delta \rho_{ij}^{st}$$ (3)

where $\Delta l_{j}^{st}=l_{j}^{t}-l_{j}^{s}$, $\Delta m_{j}^{st}=m_{j}^{t}-m_{j}^{s}$, $\Delta n_{j}^{st}=n_{j}^{t}-n_{j}^{s}$.

The process of estimating the carrier phase perimeter ambiguity consists of two main steps [14,15]. The first step is decorrelation, which involves integer processing of the ambiguities before the double-difference type, significantly reducing their correlation and thereby decreasing the number of ambiguity sets, thus improving the efficiency of the algorithm. The second step is ambiguity searching.

To resolve integer ambiguities in real-time kinematic positioning, the least squares method is applied. The goal is to minimize the weighted squared differences between the integer ambiguity candidates and the float estimates. Equation (4) formalizes this optimization process:


$$\overset{˘}{a}=\text{argmin}{\left(a-\hat{a}\right)}^{T}Q_{\hat{a}}^{-1}\left(a-\hat{a}\right)$$ (4)

If $Q_{\hat{a}}^{-1}\;$ is a diagonal matrix, the optimal solution $\overset{˘}{a}\;$ is equal to the directly rounded value of $\hat{a}\;$.However, fuzziness is previously correlated, $Q_{\hat{a}}^{-1}\;$ is not a diagonal matrix, and the optimal solution $\overset{˘}{a}\;$ can only be obtained by search. Since $Q_{\hat{a}}^{-1}\;$ is a weighting matrix with practical significance, for different measurements, the weights between them will differ greatly, and the search space determined from this becomes longer, which will make the search time longer. To address this challenge, a Z-transform is applied to decorrelate ambiguities [16], as shown in Equation (5):


$$\begin{array}{c} \left\{ \begin{array}{c} z=Z^{T}a\\ \hat{z}=Z^{T}\hat{a}\\ Q_{\hat{z}}=Z^{T}Q_{\hat{a}}Z \end{array}\right.\; \end{array}$$ (5)

where z and $Q_{\hat{z}}\;$ are the vectors after the integer transformation of a  and $Q_{\hat{a}}\;$ respectively. To ensure that the transformation between a and z is an integer transformation, $z\in {\mathbb{Z}}^{n},Z\in {\mathbb{Z}}^{n\times n}$. Therefore, equation (4) can be transformed into equation (6) through equation (5):


$$\overset{˘}{z}=\text{arg}\min_{z\in {\mathbb{Z}}^{n}}{\left(z-\hat{z}\right)}^{T}Q_{\hat{z}}^{-1}\left(z-\hat{z}\right)$$ (6)

By decomposing $Q_{\hat{z}\;}$,we have $Q_{\hat{z}\;}=L^{T}DL$.Thus, we obtained equation (7) through equation (6) as:


$$F(z)={\left(z-\hat{z}\right)}^{T}L^{-1}D^{-1}L^{-T}\left(z-\hat{z}\right)\leq \chi^{2}$$ (7)

where L is a lower triangular matrix, and D is a diagonal matrix with all diagonal elements greater than 0. The purpose of decomposing $Q_{\hat{z}\;}$ is to diagonalize $Q_{\hat{z}\;}$ as much as possible, which means minimizing the off-diagonal elements in L. Additionally, the decomposition of $Q_{\hat{z}\;}$ allows the diagonal elements in *D* to be rearranged in ascending or descending order. The size of the search ellipsoid is determined by $\chi^{2}$.

The method for resolving ambiguities involves searching within the ellipsoidal space defined by $\chi^{2}\;$.However, the value of $\chi^{2}\;$ is closely related to the success of fixing the ambiguities. When the value of ${\;\chi }^{2}\;$ is relatively small, it is possible that the optimal integer solution cannot be obtained, resulting in a failure to fix the ambiguities; When the value of$\;\chi^{2}\;$is large, the search time increases and efficiency decreases. Therefore, it is essential to choose an appropriate ${\;\chi }^{2}$ value.

After decorrelation processing, the overall search space for ambiguities has been significantly improved; however, it still contains a considerable number of candidate values [17,18]. It is necessary to further refine the search space.

From the derivation of the above formula, the size of the search ellipsoid is expressed as equation (8):


$${\left(z-\hat{z}\right)}^{T}Q_{\hat{z}}^{-1}\left(z-\hat{z}\right)\leq \chi^{2}$$ (8)

For an n-dimensional fuzzy search space, its ellipsoid volume can be quantified as equation (9):


$$E_{n}={\;\chi }^{n}\sqrt{\left|Q_{\hat{a}}\right|}V_{n}$$ (9)

where $\;\left|Q_{\hat{a}}\right|\;$ denotes the determinant of the covariance matrix $Q_{\hat{a}}\;$ and ${\;V}_{n}\;$ denotes the volume function and is defined as:


$$V_{n}=\frac{2}{n}\frac{\pi^{\frac{n}{2}}}{\Gamma \left(\frac{2}{n}\right)}$$ (10)

where Γ(·) denotes the gamma function, which is defined as:


$$\Gamma (z)=\int_{0}^{\infty }t^{z-1}e^{-t}dt=\frac{e^{-\gamma z}}{z}\prod_{k=1}^{\infty }{\left(1+\frac{z}{k}\right)}^{-1}e^{z\text{/}k},z>0$$ (11)

It has been shown in the literature that the volume of an n-dimensional ellipsoid is closely related and approximately equal to the integer [19–21] values it contains, as shown in Equation (12):


$$p=\text{nint}(E_{n})$$ (12)

where nint (·) denotes the integer closest to a certain value. Calculate $\;\chi^{2}\;$ by equation (7) and then find the appropriate fuzzy degree group in the search space determined by $\;\chi^{2}$.

After successfully fixing the whole week’s fuzziness, the next step is to check the fuzziness to make sure that the fixed fuzziness is correct. This prevents the problem of low accuracy even though the fuzziness has been fixed successfully. To validate the reliability of the ambiguity-fixed solution, the validation criterion defined in Equation (13) is applied to verify the precision:


$$R=\frac{{\left({\overset{˘}{z}}_{2}-\hat{z}\right)}^{T}Q_{\hat{z}}^{-1}\left({\overset{˘}{z}}_{2}-\hat{z}\right)}{{\left(\overset{˘}{z}-\hat{z}\right)}^{T}Q_{\hat{z}}^{-1}\left(\overset{˘}{z}-\hat{z}\right)}\geq R_{thres}$$ (13)

where $\;{\overset{˘}{z}}_{2}\;$ represents the suboptimal integer ambiguity, which is the ambiguity value just below the optimal one. $\overset{˘}{z}\;$ denotes the optimal integer ambiguity, and $R_{thres}\;$ is the set threshold, typically taken to be 2 or 3. Then the whole week fuzzy degree that passes the test is inverted to get the whole week fuzzy degree before the descending correlation, and then the other unknown parameters are solved.

In order to improve the performance of integer Gaussian transform, sorting plus greedy selection [18], Greedy selection strategy and lazy transformation strategy, are used. The greedy selection strategy reduces the number of permutations, while the lazy transformation strategy avoids unnecessary integer Gaussian transformations, thereby decreasing the decorrelation time and improving the algorithm’s efficiency.

### 2.2. Intelligent early warning modeling

**Fig 1:**
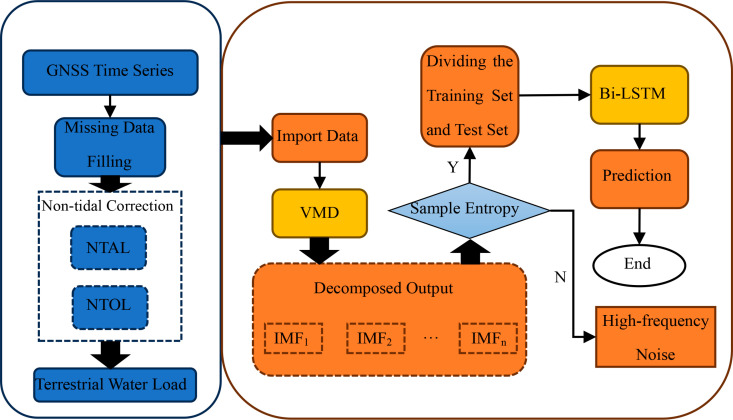
VMD-BiLSTM intelligent early-warning technical flow.

Using the excellent decomposition capability of Variational Mode Decomposition (VMD) and the powerful learning ability of Bi-LSTM, we modeled the complex displacement time series monitored by BDS/GNSS. First, VMD was applied to perform dimensionality reduction on the original time series. The IMFs (Intrinsic Mode Functions) were classified into high, medium, and low frequencies using the run-length test method, representing fluctuation components, short-cycle components, and long-trend components of the original time series, respectively. Temporal-spatial deep learning was then applied to each group of components, followed by recombination of the components. The technical route of the intelligent early-warning model is shown in Fig 1.

It is assumed that the time series signal is decomposed into k IMF components [22], and a constrained variational model is established, as shown in equation (14) and (15):


$$\;\begin{array}{c} \min\\ \text{\textbackslash{}\{}u_{k}\text{\textbackslash{}},\{\}\omega_{k}\text{\textbackslash{}}\} \end{array}\left\{\sum_{k}{\left\|\partial_{t}\left[\left(\delta (t)+\frac{j}{\pi t}\right)*u_{k}(t)\right]e^{-j\omega_{k}t}\right\|}_{2}^{2}\right\}\;(\sum_{k}u_{k}=f)$$ (14)

$$L(\left\{u_{k}\text{\textbackslash{}},\{\}\omega_{k}\text{\textbackslash{}},\}\;\lambda \right)=\alpha \sum_{k}{\left\|\partial_{t}\left[\left(\delta (t)+\frac{j}{\pi t}\right)*u_{k}(t)\right]e^{-j\omega_{k}t}\right\|}_{2}^{2}+{\left\|f(t)-\sum_{k}u_{k}(t)\right\|}_{2}^{2}+\left\langle \lambda (t),f(t)-\sum_{k}u_{k}(t)\right\rangle$$ (15)

where k represents the number of intrinsic mode components, $u_{k}$ is the set of all modes, $\omega_{k}$ is the actual center frequency of each mode, $\partial_{t}$ represents the gradient operation, δ(t) represents the shock function, $j^{2}=-1$, t is the time, $e^{-j\omega_{k}t}$ is the estimated center frequency of each analytic signal, ‖·‖ represents finding the $L_{2}$ norm, ∑  is the sum of all model components. ⟨⟩ is the inner product operator. The original minimization problem is changed into the present one by using alternating direction multiplier algorithm to find the optimal solution by iterative updating.

Sample Entropy (SE) is an indicator used to measure the complexity of time series or signals, which can be used to describe the degree of randomness and irregularity of data. It is based on the concept of information entropy and is used to estimate information loss and uncertainty in a sequence. The higher the SE, the greater the uncertainty and complexity in the sequence, while a lower SE indicates a more regular and predictable sequence. In this article, decisions will be made based on the SE value for the selection of IMF components, as shown in equation (16):


$$SE=\;\frac{-ln[A(m,r)]}{-ln[A(m+1,r)]}$$ (16)

where A(m, r) represents the proportion of similar subsequences given a subsequence length m and a threshold r (usually 0.1~0.25 times the subsequence standard deviation).In order to distinguish between noise components and effective signals, this paper sets the threshold of SE to 0.5 based on the idea of grey wolf optimization and extensive experiments [22]. This approach can retain low-frequency components with SE values less than the threshold in all IMF components, and synthesize them into denoised signals, which can effectively filter out noise in deformation monitoring time series data.

Bi-LSTM is a deep learning mode, which performs computations in both the forward and backward directions simultaneously at each step. The detail description on the method can be found in the references [23–25].

## 3. Results

### 3.1. Combined BDS/GNSS test results

**Fig 2:**
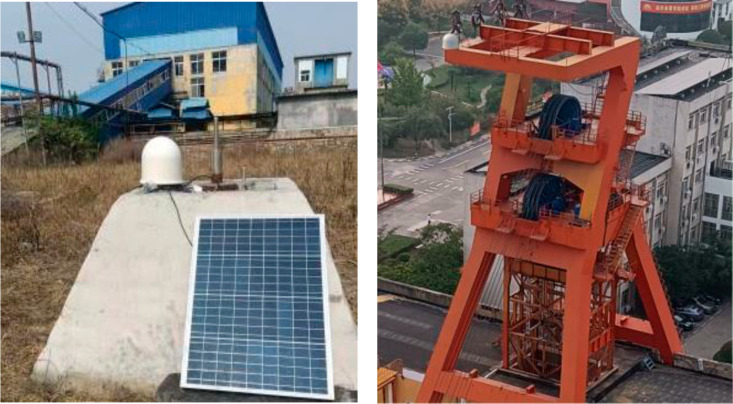
Deformation monitoring system in mining area including reference station and monitoring station. (The left is the reference station, a highly stable bedrock benchmark cement reinforced concrete structure. The right is the monitoring station located on the wellhead, representing the deformation of the wellhead).

In the experiment, we choose a high stable site based on bedrock observation as the reference station. The monitoring sites are distributed deformation area, representing the deformation characteristic of wellbore (see Fig 2). The data used in this experiment consists of all day observation with a sampling rate of 1 HZ. The positioning strategy adopts the four-system combination solutions of GPS/BDS/GALILEO/GLONASS(G/C/E/R). During data processing, the satellites G25, R05, R15, C01, C02, C03, C04, and C05 were excluded. The main reason for excluding satellites G25, R05, and R15 is that when these satellites were included in the computation, the results became float solutions with poor accuracy. Therefore, these three satellites were excluded. Upon reviewing the original observation data, it was found that R05 had a satellite elevation angle greater than 15° at 04:13, R15 at 04:19, and G25 at 04:21, but the elevation angles were still relatively low, leading to overall poor calculation accuracy. The main reason for excluding satellites C01, C02, C03, C04, and C05 is that these satellites are GEO satellites, meaning they are geostationary satellites. They generally remain in a state of slight oscillation on their orbits, resulting in much larger orbital errors compared to MEO satellites, and they do not exhibit spatial variation trends (remaining relatively stationary with respect to the Earth). Therefore, when there are enough positioning satellites available, it is advisable to consider not including these satellites in the calculations. In addition, a comparative analysis was conducted on the performance of BDS-3 and BDS-2 in terms of observed carrier-to-noise ratio, multipath effects, and other factors. The results indicate that BDS-3 effectively addresses the code bias issues present in BDS-2, and it was also found that the next-generation atomic clocks on BDS-3 exhibit higher stability compared to those on BDS-2.

The number of the visula satellites and the DOP values for the different system combination are shown in Table 1. It can be seen clearly that as the number of combined systems increases, the number of satellites gradually increases, and the PDOP, HDOP, and VDOP values gradually decrease. Furthermore, the number of satellites in the four-system combination is 30 or more, with PDOP, HDOP, and VDOP values all less than 1.0.

**Table 1:** Visual satellites number and DOP values for different system combination (G. C, E, and R represents GPS, BDS, GALILEO, GLONASS respectively, and “/”represents the combined positioning of multiple systems).

Systems	Average number of satellites	Average DOP		
PDOP	HDOP	VDOP
G	8	2.37	1.18	2.05
G/C	23	1.23	0.63	1.05
G/C/E	29	1.06	0.54	0.91
G/C/E/R	35	0.95	0.49	0.82

**Fig 3:**
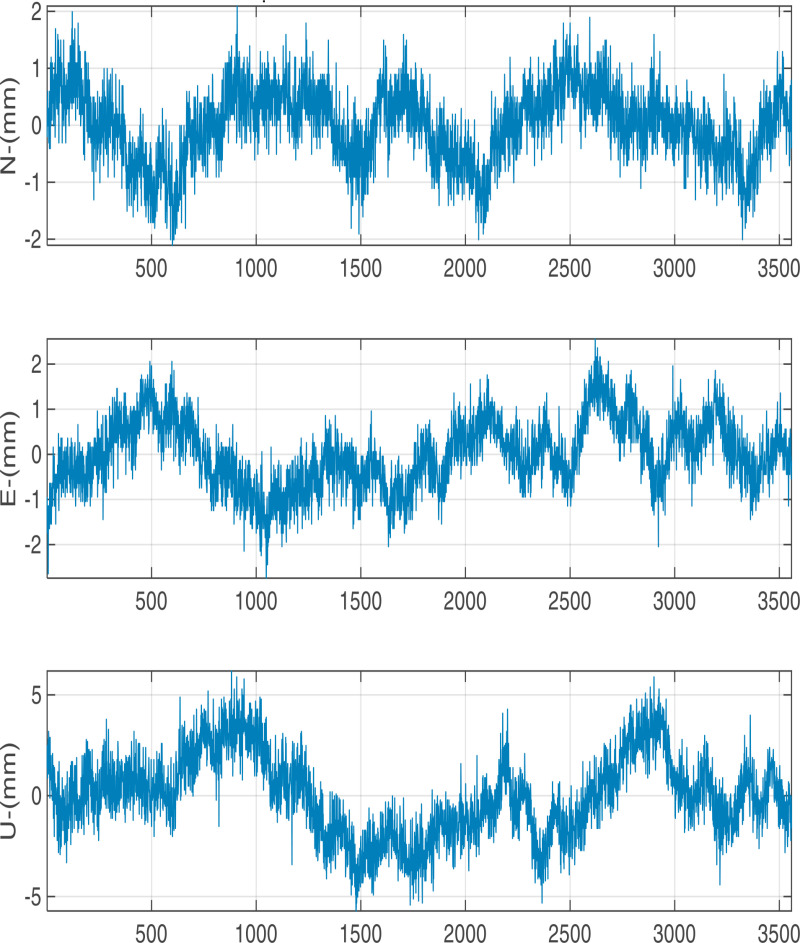
GPS/BDS/GALILEO/GLONASS monitoring results.

The positioning results in one hour with a sampling interval of 1s for G/C/E/R are shown in Fig 3. Obviously, the displacement fluctuation is largely concentrated in ±2 mm in horizontal (N, E) direction and ±5 mm in vertical direction.

Table 2 show the statistic standard deviation (STD) of the whole displacement time series from different satellite positioning system. STD for single-system positioning GPS is better than 2 mm in the horizontal direction, and 3 mm in the U direction while the solution accuracy for multi-system combination positioning is better than 1 mm in the horizontal direction, and 2 mm in the U direction. This indicates that as the number of navigation systems increases and the number of satellites rises, the internal positioning accuracy of multi-system combination positioning significantly is improved, demonstrating the advantages of multi-system combination positioning. From Table 1, it also can be found that the positioning accuracy for G/C, G/C/E, and G/C/E/R are quite close. This is primarily because the observation satellites from GPS and BDS have been sufficiently abundant, so that the satellite geometric configurations are very similar for the three-systems or four-systems combination solution. This indicates that all satellite positioning systems achieved the expected high-precision levels during the computation process. This result indicates that the computation strategies established for each system are correct and effective, successfully avoiding a significant number of unresolved cycle slips and errors in the initial station coordinates, thereby ensuring the accuracy and reliability of the positioning results.

**Table 2:** Statistic STD values of different satellite systems. G. C, E, and R represent GPS, BDS, GALILEO, GLONASS, respectively. “/ ” represents the combination of different satellite system).

System	STD/mm		
E component	N component	U component
G	1.4	1.9	2.6
G/C	0.8	1.0	2.2
G/C/E	0.7	0.8	2.0
G/C/E/R	0.7	0.8	2.0

### 3.2. Comparison of monitoring results from different baseline length

**Fig. 4:**
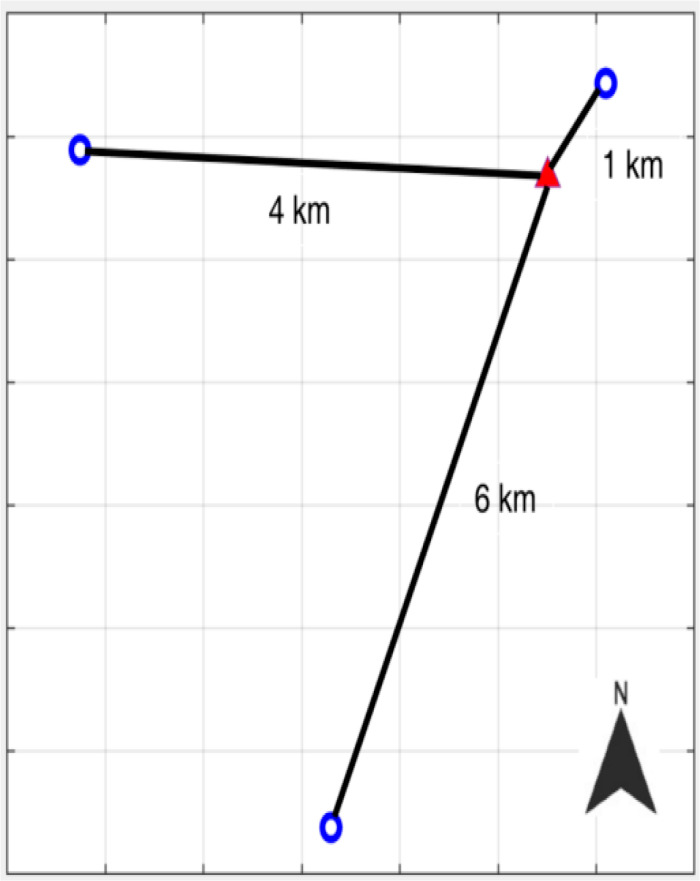
Point distribution. Red triangle symbol is used as the reference station, and blue circle symbols are used as three monitoring stations. Four stations construct baselines net with three different distances of bout 1 km, 4 km, and 6 km, respectively.

A stable station is chosen as the reference station and three monitoring stations at different distances are adopt to form three baselines groups with different distances of about 1 km, 4 km, and 6 km. The point distribution are shown in Fig 4. The purpose is to calculate the displacement change of the monitoring station relative to the stable reference station. It represents the actual deformation of monitoring sites in absolute East, North, Up direction.

**Fig 5:**
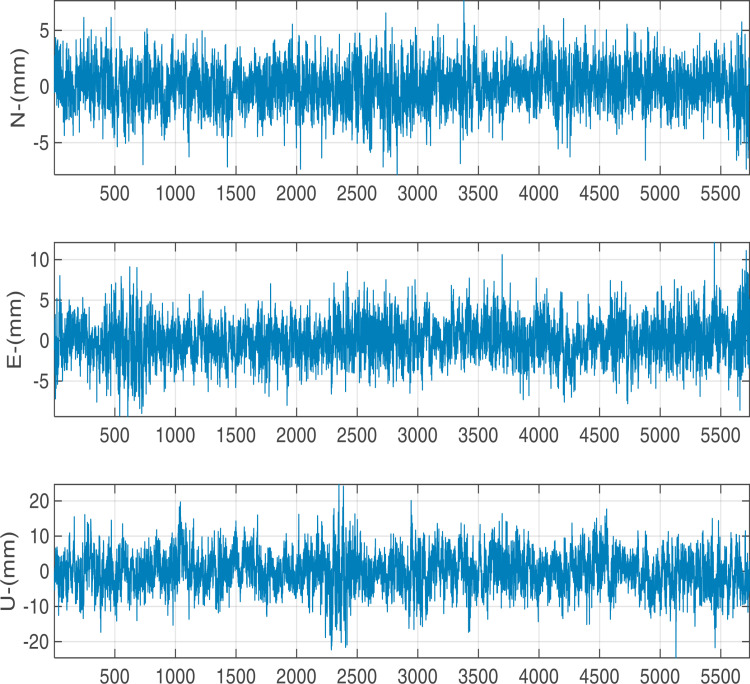
Positioning results with 1 km baseline length.

**Fig 6:**
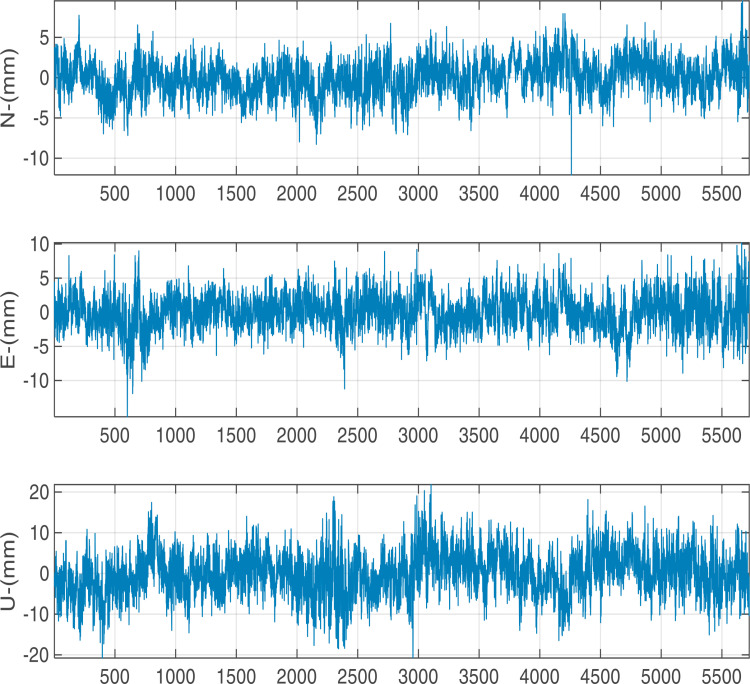
Positioning results with 4 km baseline length.

**Fig 7:**
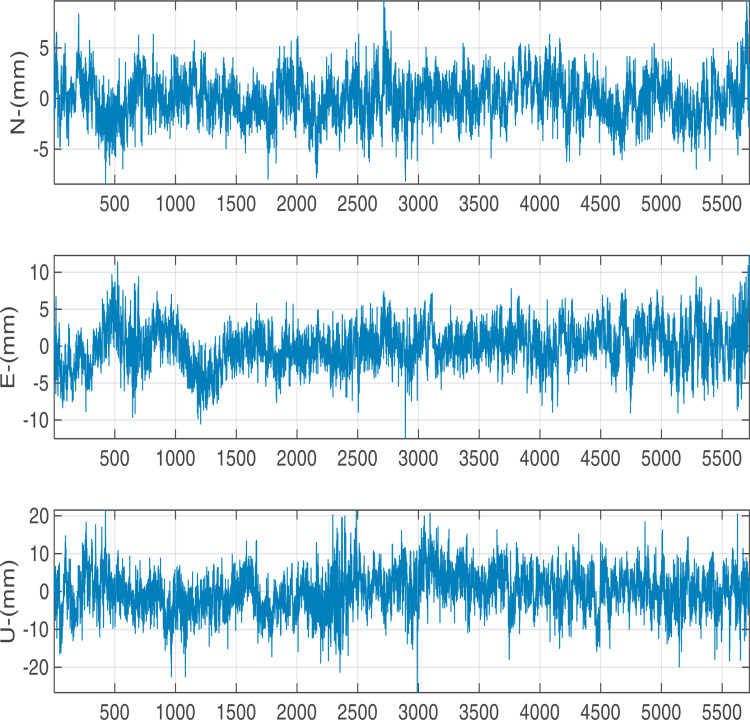
Positioning results with 6 km baseline length.

We utilized 5760 epochs per day with a 15 seconds sampling interval for testing the precision with different baseline vectors. The calculated displacement time series in N, E, U components are shown in Figs 5–7. At different baseline, there are significant periodic fluctuations in N, E and U directions, especially in U direction: the amplitude of the fluctuation in N,E direction is largely concentrated in ±5 mm and the amplitude in U direction is largely concentrated in ±10 mm.

The calculated STD values are presented in Table 3. With the baseline length increases from 1 to 6 kilometers, the accuracy in the E, N, and U directions decreases by 15.8%, 16.0%, and 5.6%, respectively. Within the range of 6 km, the horizontal accuracy can remain within 3 mm and the vertical accuracy is better than 6 mm.

**Table 3:** Accuracy statistics for different baseline lengths.

Baseline length	STD/mm		
E component	N component	U component
1 km	1.9	2.5	5.4
4 km	2.2	2.6	5.5
6 km	2.2	2.9	5.7

### 3.3. Bi-LSTM intelligent early-warning

Based on the calculated displacement data with 81 epochs with a 15 seconds sampling interval, we validate the effectiveness of the early warning model by separating training set for machine learning, testing set for validation and future forecasting, respectively.

**Fig 8:**
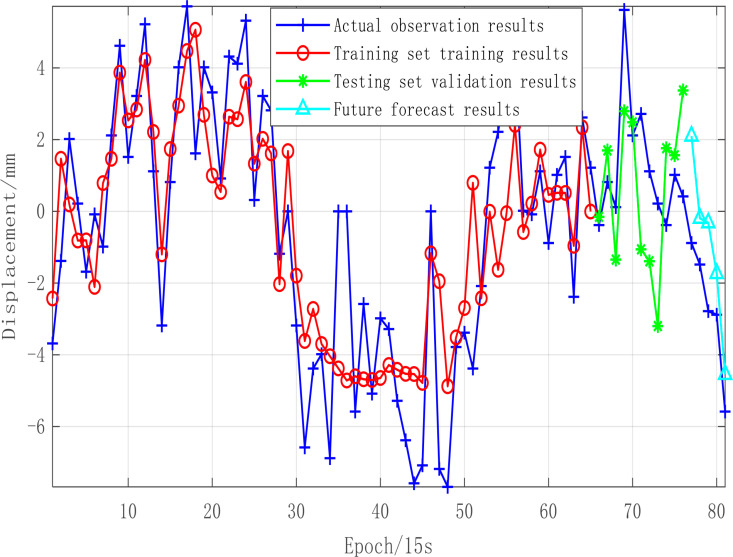
Bi-LSTM intelligent early warning model results.

The results are as shown in Fig 8. The trained, validated and predicted results well agree well with the observed displacement time series, showing a high degree of consistency. The Root Mean Square Errors (RMSE) are 2.3 mm, 3.1 mm and 0.9 mm, demonstrating the improved accuracy for shorter forecasting periods. As more observational data is accumulated, the model’s accuracy will further improve, facilitating the development of a more precise forecasting model. This analysis shows that the current observational data can reliably predict displacement changes within the upcoming 5 epochs, meeting the requirements for deformation anomaly monitoring and early warning.

## 4. Discussion

### 4.1. Performance

This study processed real measurement data from a BDS/GNSS station in a specific mining area, obtaining deformation monitoring results for different baseline lengths across various satellite system combinations. The monitoring stations are distributed in various mines and ventilation openings, open and continuous to ensure monitoring accuracy. The experiment shows that the positioning accuracy of the BDS/GNSS multi-system combination is optimal, with varying degrees of improvement in E,N,U direction accuracy compared to the single GPS system. The increase in baseline length has different effects on the positioning results, providing scientific data references for borehole deformation monitoring in mining areas. The deformation in the mining area is slow and it undergo a process of gradual changes with the increase of mining resource exploitation. In this study, the deformation due to mining activities is about a few centimeters per year. Therefore, the displacement change in one day is negligible. Through long-term observation, anomalous changes in trend can be observed. If the difference is greater than the observation error or greater than three times STD, a warning will be issued. In mining area, rock movement caused by nearby mining activities is main reason of displacement generation. It can affect the foundation of the derrick, causing uneven settlement, which can tilt the derrick or partially deform it. It is necessary to consider the time factor, long-term use of the derrick may cause the stiffness to decline due to material fatigue, corrosion and other factors, and the deformation will gradually accumulate. At this time, the deformation may appear as a slow plastic deformation, rather than just an elastic deformation. In terms of safety, if the deformation of the derrick exceeds the allowable value, it may affect the normal operation of the lifting system, and even lead to structural failure, resulting in safety accidents. Therefore, the study of deformation anomaly is very important for regularly monitoring and detect deformation characteristics during maintenance, and to strengthen or repair in time. In subsequent research, we will dynamically optimize warning thresholds based on real-time monitoring data to effectively detect potential catastrophic events.

The LSTM model can learn the contextual relationships within the GNSS time series, while the Bi-LSTM model can capture dependencies in both directions—from the beginning and end of the GNSS time series, allowing for a more comprehensive learning of temporal dependencies. In this study, VMD decomposition was applied before Bi-LSTM prediction to remove most high-frequency noise, making the primary components of the time series clearer and significantly improving the iterative efficiency and predictive accuracy of the Bi-LSTM model. As shown in Fig 8, the model effectively learns the characteristic changes in the GNSS time series within the training set and demonstrates excellent predictive performance on the test set. Additionally, after verifying model reliability through test set predictions, the trained model was used to predict the next 5 epochs, showing strong consistency with the observed values.

### 4.2. Limitations and extensions

Our work in the manuscript mainly focus on the comprehensive application and integration of these methods to address challenges in GNSS high-precision deformation monitoring and intelligent early warning for wellbore. Results experiment show that the strategy is successful, we have been developing an automated data resolution platform. However, it requires some computational costs. First, computational costs, including receiving signals from multiple satellites, processing the raw data to calculate positions, applying corrections for Real-Time Kinematic (RTK) accuracy, handling data transmission, and possibly running algorithms for error detection or predictive analytics. Especially, RTK processing, which is used for high-precision applications, involves resolving integer ambiguities in the carrier phase measurements. That’s a computationally intensive task. Data transmission is another aspect. Real-time systems need to send and receive data continuously so that the system can’t afford delays, which means the algorithms must be efficient. If the system is using a network of reference stations, aggregating that data and broadcasting corrections (e.g., NTRIP-Networked Transport of RTCM via Internet Protocol) would require bandwidth. Also, if the system is cloud-based, there might be costs associated with data storage and processing in the cloud. If you’re monitoring a large number of points, the system needs to handle multiple data streams simultaneously, which increases computational demands. Other limitations might be related to the environment. For example, in urban canyons with tall buildings, GNSS performance is notoriously poor. Future research should consider the integration with other systems. Combing GNSS with IMUs (Inertial Measurement Units) by the fusion algorithms like Kalman filters can provide position data when GNSS signals are lost.

The displacement time series are usually suffered from the influence of temperature change and hydrological loading deformation, however, these exhibit the characteristic of the periodic or regular characteristics, distinguishable from structural deformation of the headframe. Also, wind, earthquake and other natural factors will result in the deformation. If these factors can be considered and corrected, the monitoring level of wellbore will get the further improvement. It will be our further research in future. In the case of real-time monitoring, the monitoring accuracy of the horizontal direction is better than 3 mm, and the vertical direction is better than 6 mm. In case of quasi-real-time monitoring, the monitoring accuracy will be further improved. The solution of 1h observation is better than 2 mm in horizontal direction, better than 3 mm in the vertical direction. Furthermore, the precision values only represent the raw solution accuracy. Through post-processing filtering, the horizontal precision can be improved to 1–2 mm, while the vertical precision reaches 3–4 mm, which can meet the needs of monitoring and early warning for wellbore. Our future research will focus on algorithm optimization for error reduction in all three coordinate components to satisfy the high-precision monitoring requirements. We will also improve the monitoring accuracy by filtering algorithms and further optimize the warning model using machine learning.

## 5. Conclusion

This paper analyzes the effectiveness of BDS/GNSS multi-system combinations in high-precision deformation monitoring and early warning for wellbores in mining areas. The solution accuracy of BDS/GNSS multi-system combination positioning is better than 2 mm in the E, N, and U directions, demonstrating higher precision compared to single GPS system positioning. This indicates that as the number of navigation systems increases and the number of visible satellites rises, the results and precision of multi-system combination positioning significantly improve, demonstrating the advantages of multi-system combinations in high-precision deformation monitoring in complex environments. As the baseline length varies from 1 to 6 km, the monitoring accuracy is affected to different degrees, however, the horizontal accuracy in the E and N directions remains within 3 mm, while the vertical accuracy in the U direction stays within 6 mm for baselines up to 6 km. This is the accuracy of direct calculation. After post-processing filtering, the horizontal EN direction can be improved to 1–2 mm, and the U direction can be improved by 3–4 mm. Based on high-precision monitoring solution results, a mining intelligent early warning model was established by combining modal decomposition using VMD with deep learning Bi-LSTM. This model can effectively capture the complex nonlinear changes in deformation monitoring time series and flexibly adapt to the conditions of actual sites and various influencing factors. The high-precision monitoring and early warning model discussed in this paper can be applied to other high-precision deformation monitoring fields, holding significant importance for the promotion of high-precision BDS applications.
